# Proteomics and Bioinformatics Profiles of Human Mesothelial Cell Line MeT-5A

**DOI:** 10.3390/proteomes14010002

**Published:** 2026-01-04

**Authors:** Rachel L. Watkin, Avedis A. Kazanjian, Jennifer R. Damicis, Elizabeth Yohannes

**Affiliations:** 1 Department of OB/GYN, Madigan Army Medical Center, 9040 Jackson Ave, Tacoma, WA 98431, USA; 2 Department of Clinical Investigation, Madigan Army Medical Center, 9040 Jackson Ave, Tacoma, WA 98431, USA

**Keywords:** the mesothelial MeT-5A cell line proteome, cellular proteome, conditioned medium proteome, LC-MS, GO annotation

## Abstract

**Background**: Despite existing proteomics studies of other cell types, a comprehensive proteome of mesothelial cells has not been characterized. This study establishes a crucial baseline proteome for mesothelial cells to better understand their fundamental bioprocesses in healthy and injured states. **Methods**: Using mass spectrometry-based shotgun proteomics, we characterized the cellular fraction (CF) and conditioned medium (CM) proteomes of mesothelial cell line MeT-5A. The datasets were analyzed for Gene Ontology (GO) terms and canonical pathway enrichments to identify biological themes. **Results**: Our analysis identified 5087 protein groups, including 1532 shared proteins, 3122 unique to the CF and 433 exclusive to the CM. GO annotation revealed distinct functional enrichment profiles, reflecting the differing roles of intracellular and secreted proteins. While intracellular proteins were linked to core cellular functions, the extracellular proteome was enriched for signaling and cell-to-cell interaction pathways. The proteins shared by both compartments provided an integrated view of the molecular coordination between the cellular and extracellular environments. **Conclusions**: This study provides the first comprehensive baseline proteome for mesothelial cells and their secreted medium, offering a vital resource for future investigations into the mesothelium, particularly in the context of disease or injury.

## 1. Introduction

The mesothelium is composed of a single, continuous layer of specialized epithelial cells (mesothelial cells) that line the body’s coelomic (serous) cavities (pleura, peritoneum, pericardium) and cover the internal organs within these cavities. It is a dynamic structure and plays key roles in fluid and cell transport, inflammation and healing, immune response and regulation, promoting both the deposition and clearance of fibrin, antigen presentation, and tumor cell adhesion and dissemination [1,2]. Much of the demonstrated mesothelium functions and responses to challenges are based on in vitro studies using primary and/or immortalized cells such as MeT-5A as a proxy to mesothelium and have been shown to involve numerous proteins [2,3]. For example, mesothelium defense and/or repair mechanisms to injuries have shown to involve proteins that can either (1) exacerbate an inflammatory response (cytokines); (2) recruit professional phagocytic cells (chemokines); (3) reorganize the cytoskeleton and cell migration to support self-recovery; (4) destroy microbes directly with or without inducing a secondary immune response (certain AMPs, glycocalyx); or (5) mediate critical roles in driving un-healthy tissue repair, such as adhesions between organs [4,5,6,7,8,9,10,11]. Therefore, deciphering the human mesothelial cell proteome is vital to fully understand the structure, function, and mechanism(s) of protection elicited when mesothelial cells are intact, as well as when they are breached.

Cell lines have been indispensable for biological research for several decades. Immortalized cells provide greater accessibility and reduce inter-donor variability compared to primary cells, and the findings derived from their use are widely published [12,13,14]. We acknowledge that some research findings using immortalized cells may not completely reflect those from primary cells, due to phenotypic characteristics or protein profiles that are not fully representative of actual primary tissue [12,14]. However, these issues are less pronounced for many applications and can be mitigated by carefully establishing reference proteome profiles and validating findings with primary cells where appropriate [15]. Baseline proteomes of cell lines provide a solid foundation for studies examining their dynamic responses in in vitro experiments, and serve as an inventory of their building blocks. Knowledge of a cell line proteome dataset with associated abundance profiles can be harnessed to establish quantification standards for proteins of interest and inform the selection of appropriate experimental platforms.

Thus, there has been growing interest in exploring the system-wide view of cell line proteomes using advanced proteomics technologies. While a growing body of proteomics studies exists for various cell lines, to our knowledge, there remains a scarcity of studies specifically focused on identifying and annotating the baseline proteome of mesothelial cells and/or their conditioned medium [16,17,18,19].

The goal of the present study was to employ high-throughput proteomics and bioinformatics approaches to identify and characterize the proteome of mesothelial cell line MeT-5A, and the MeT-5A-conditioned medium. We analyzed and identified the proteome of the mesothelial cell lysate and conditioned medium using high-throughput liquid chromatography (LC) coupled with tandem mass spectrometry (MS/MS). We annotated the proteome based on Gene Ontology (GO) terms that described the cellular component, the molecular function, the biological process, and their membership on canonical pathways, as well as participation in the InnateDB, using bioinformatics tools.

## 2. Materials and Methods

The mesothelial cell line MeT-5A, obtained from the American Type Culture Collection (ATCC) and originally isolated from the pleural fluids of a non-cancerous individual, was used as the source of material for this study.

### 2.1. Cell Culture and Proteome Preparation

We cultured three technical replicates of MeT-5A cells (ATCC, Manassas, VA, USA) at 37 °C and 5% CO_2_ in modified optimum medium: containing Medium-199 supplemented with 1% antibiotic-antimycotic solution, 10 ng/mL epidermal growth factor, 400 nM hydrocortisone, 870 mM insulin, 0.3% Trace Elements B and 10% FBS. When MeT-5A monolayers grew to 80–90% confluence, we pipetted out the growth medium without disturbing the monolayers and harvested the cells into protein Lobind Eppendorf tubes by trypsinization of the monolayer. We then pelleted the cell by centrifugation at 300× *g*, 4 °C for 10 min, separated and discarded the supernatant. The cells were cleaned and pelleted twice by re-suspension in phosphate-buffered saline (PBS) treated with protease and halt phosphatase inhibitor cocktail (Thermo Fisher Scientific, Waltham, MA, USA). We lysed the cells and processed using an established filter-aided sample preparation (FASP) protocol with minor modification [20]. Briefly, we pelleted 150 µL aliquot of cell suspension from each technical replicate (*n* = 3) by centrifugation at 300× *g* for 10 min at 4 °C to remove the final PBS rinse, re-suspended and lysed in lyses buffer containing 4% sodium dodecyl sulfate (SDS), 100 mM dithiothreitol (DTT), 1x halt protease and phosphatase inhibitor cocktail, and 25 mM TRIZMA, pH = 8 and at 90 °C for 5 min. We homogenized the cells at room temperature for 20 s and repeated this 3 times with a 20 min incubation period on ice between each homogenization. We cleared the cell lysate from the cell debris by centrifugation at 16,000× *g* for 10 min at 4 °C. We then carefully collected cleared supernatant, transferred it into a protein LoBind tube, and determined total protein content using reducing agent compatible BCA Protein Assay Kit (Thermo Fisher Scientific, Waltham, MA, USA). We mixed about 100 µg of lysate with 200 µL of 8 M urea, 50 mM ammonium bicarbonate in a 3 kDa filter unit (Millipore Sigma, Burlington, MA, USA) spun at 14,000× *g*, for 20 min at room temperature to remove SDS. After adding 50 mM iodoacetamide (IAA) and incubation for 1 h at room temperature in the dark to carboamidomethylate the cysteine residues, we spun the unit at 14,000× *g*, for 20 min at room temperature. We depleted and washed the SDS and IAA through the 3 kDa filter by adding 8 M urea with 50 mM ammonium bicarbonate and spun the unit twice and followed by 50 mM bicarbonate wash. We diluted the protein with 50 mM ammonium bicarbonate and digested it with sequencing grade trypsin (Promega, Madison, WI, USA) at an estimated 1:50 enzyme to protein ratio for overnight at 37 °C with gentle shaking. We collected the digest and concentrated with speed vacuum. We acidified and adjusted the volume of digest with 0.1% formic acid.

### 2.2. Conditioned Medium Proteome Preparation

We cultured three technical replicates of MeT-5A cells (ATCC, Manassas, VA, USA) at 37 °C and 5% CO_2_ in modified optimum medium. When MeT-5A monolayers grown to 80–90% confluence, we trypsinized the monolayer, re-seeded nine technical replicates of cells and continued to culture the cells to confluent monolayers in optimum medium. We replaced the medium with serum free optimum medium and cultured for additional 24, 48, and 72 h (*n* = 3, per time point). We pipetted conditioned medium at each time point without disturbing the cell monolayer, transferred into conical flasks, cleared it from cell debris by consecutive centrifugation: first at 300× *g* for 10 min, collected supernatant and re-centrifuged at 2000× *g* for 20 min at 4 °C. We then collected the supernatant, transferred and concentrated on 3 kDa filter at 4 °C. After normalizing the proteome via BCA assay, we proceed with the digestion and peptidome processing steps as described above.

### 2.3. Liquid Chromatography

Liquid chromatographic analysis of tryptic peptides from cellular lysates (*n* = 3) and conditioned medium (*n* = 9) were performed with the Acquity UPLC M-class system (Waters, Milford, MA, USA) coupled with Q-Exactive HFX tandem mass spectrometer (Thermo Fisher Scientific, Waltham, MA, USA). For peptide separation, each injection (about 600 ng based on protein concentration) of each sample was loaded onto a trap column (nanoEase, 100 Å, 5 µm, 180 µm × 20 mm, Waters, Milford, MA, USA) with 0.1% formic acid at 0.3 µL/min then eluted onto an analytical column (nanoEase M/Z Peptide BEH C18, 130 Å, 1.7 µm, 75 µm × 150 mm, Waters, Milford, MA, USA) with an 180 min gradient of 5–30% acetonitrile, 0.1% formic acid at 0.3 µL/min followed by column wash and re-equilibration for a total injection run time of 203 min.

### 2.4. Mass Spectrometry

Mass spectrum data were acquired for pre resolved peptidomes from cell lysate (*n* = 3) and from conditioned medium (*n* = 9) on the Q-Exactive HFX in a data-dependent acquisition (DDA) with a top 10 configuration, auto dynamic exclusion. Precursor spectra were collected from 380 to 1800 *m*/*z* at 120,000 resolution (AGC target of 3 × 10^6^, max IT of 50 ms). Peptide fragmentation was performed with the HCD and MS/MS scans were collected on +2 to +6 precursors at 30,000 resolution (AGC target of 1 × 10^6^, max IT of 50 ms) with an isolation width of 1.6 *m*/*z* with a NCE of 30.

### 2.5. Protein Identification and Data Search Parameters

Raw data files were processed using Proteome Discoverer, version 2.4 (Thermo Fisher Scientific, Waltham, MA, USA). Peak lists from technical replicates were extracted and merged separately for the cell lysate samples (*n* = 3 replicates) and the conditioned medium samples (*n* = 9 replicates, across three time points). The merged peak-list files were searched against the human Swiss-Prot FASTA database (20,341 sequences). The database version downloaded from UniProt on 2 August 2024, was used for the cell lysate analysis, while the version from 28 February 2025, was used for the conditioned medium analysis. The general workflow used has been described in detail previously [21,22]. The peptide-spectrum match was considered correct if it achieved an estimated q-value (adjusted *p*-value) (minimal false discovery rate) of 0.01 or less. For protein identification, a minimum of one unique peptide with delta Cn (delta correlation) ≤ 0.05 and with high confidence based on q ≤ 0.01 were utilized to ensure the protein level stringency.

### 2.6. Proteome Functional Classification Analyses

Functional enrichment and pathway analysis were performed using two bioinformatics tools. Gene Ontology (GO) term classification (cellular component, molecular function, and biological process) and protein domain enrichment were analyzed using the standalone software FunRich version 3.1.4 (http://www.funrich.org), last accessed on 5 August 2025. For this analysis, the integrated human-specific FunRich background database was used [23]. For enrichment analysis of canonical pathways, we utilized Ingenuity Pathway Analysis (IPA) software, 2025 summer release (content accessed on 7 August 2025; www.ingenuity.com) and its manually annotated canonical pathways database (Qiagen, Redwood City, CA, USA). Statistical significance for FunRich analysis was determined using the Hypergeometric distribution test, while IPA employed Fisher’s exact test. For both analyses, a Benjamini–Hochberg (BH) false discovery rate (FDR) method was applied to correct for multiple testing, yielding adjusted *p*-values.

To allow a more comprehensive assessment of the differences or similarities between the proteomes of cellular fraction and conditioned medium, we first divided the datasets into three groups including (1) all observed proteins as uniquely identified within the cellular fraction (CF), (2) all observed proteins uniquely identified within conditioned medium (CM), or (3) proteins identified in both (shared CF/CM). We then assessed proteins identified as shared CF/CM based on the ratio of abundance of each protein within CF compared to CM. We grouped proteins with log fold-change of ≥2 and <4 peptide spectrum matches (PSMs) in the CM as proteins enriched within the cell fraction and merged to the CF unique group. Conversely, proteins with log fold-change of ≤−2 and <4 PSMs in the CF were grouped as proteins enriched within condition medium and merged to the CM unique group. The remainder that did not meet these criteria were kept as the shared CF/CM group. With this approach, we have generated three datasets for functional analysis including proteins unique and enriched within CF, proteins unique and enriched within CM, and proteins present in both CF and CM or shared CF/CM. The enriched terms of cellular components, molecular functions, biological process, canonical pathways, or protein domains were considered significant if they achieved a BH adjusted *p*-value of (minimal false discovery rate) 0.05 or less.

## 3. Results and Discussion

**Figure 1:**
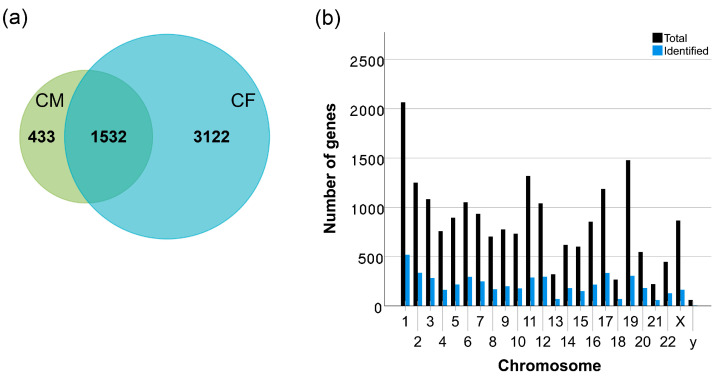
Summary of cellular and conditioned medium proteomes of MeT-5A. (**a**) Venn diagram illustrating the total number of proteins identified by high-throughput LC-MS/MS from two distinct sample types: cellular lysate and conditioned cell culture medium. The numbers within each circle represent the total number of proteins uniquely identified in either the cellular lysate (blue circle) or the conditioned medium (green circle). The overlapping region indicates the number of proteins that were identified in both the cellular and conditioned medium samples. (**b**) Distribution of identified proteins (blue bars) relative to known total protein-coding genes (black bars) across human chromosomes. This bar plot illustrates the number of proteins identified in this study compared to the total number of protein-coding genes known for each individual chromosome. The x-axis represents the individual chromosomes, while the y-axis indicates the count of protein-coding genes or proteins. Blue bars denote the count of proteins identified in this study, while black bars represent the total number of protein-coding genes on each chromosome, according to Ensembl annotations.

In this study, the experimental workflow described in the methods was employed to identify 29,002 peptide groups that mapped to 5087 protein groups in both the cellular and conditioned medium (Figure 1a). The 5087 identified proteins correspond to 5074 ensemble genes, representing approximately 25% of the revised human genome. As depicted in Figure 1b, protein identification in our study is relatively evenly distributed across the human chromosomes, with an average of about 25% of the genes identified in each chromosome. This level of coverage was achieved using long gradient separation and data-dependent acquisition (DDA), thus avoiding the time-consuming and resource-intensive fractionation steps. To create a comprehensive catalog of the normal human mesothelial cell proteome, lists of proteins identified in the cellular lysate (fraction) and in the conditioned medium are tabulated in Supplementary Material S1, Tables S1 and S2, respectively.

**Figure 2:**
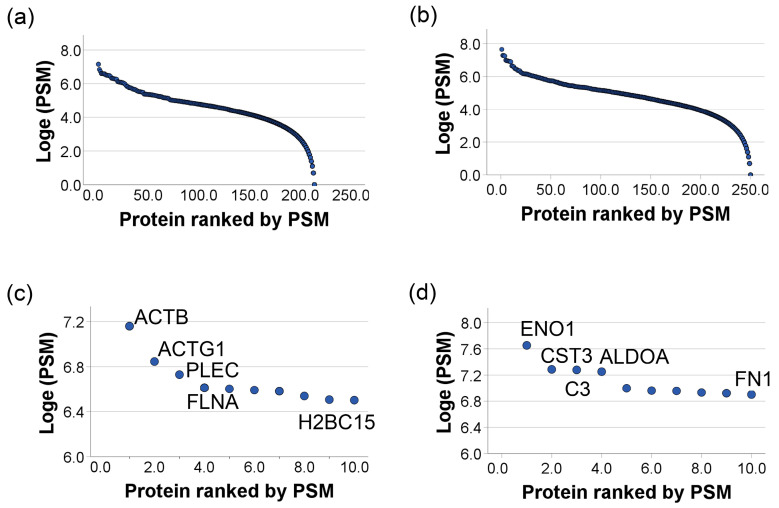
Dynamic range of cellular and conditioned medium proteomes of MeT-5A. (**a**,**b**) depict the wide dynamic range of proteins present in MeT5A cell lysate and cell conditioned medium, respectively, spanning seven orders of magnitude as shown by protein mean normalized abundance (PSM) on the Y-axis. The X-axis represents individual protein rank based on abundance. The abundance of each protein estimated based on the spectral count (defined by the total number of identified peptide spectra matched (PSM) to the protein of interest, including those redundantly identified) revealed the typical S-shaped distribution over the seven orders of dynamic range of MS signals. (**c**,**d**) show top ten most abundant proteins in the cell lysate and conditioned medium, respectively.

We have estimated the abundance for each identified protein based on spectral count defined by the total number of identified peptide spectra matched (PSM) to the protein, including spectra from redundant peptide identifications, as a proxy to protein concentration. We acknowledge that the estimated abundance is only a very rough indication of actual abundance levels. In particular, the abundance levels may be underestimated for the least abundant proteins. We established proteome dynamic ranges for cellular and conditioned medium from the mean normalized PSM of three runs for each protein (Figure 2). Aligned with known characteristics of proteome abundance distributions, the proteome abundance for both cellular (Figure 2a) and conditioned medium (Figure 2b) revealed the typical s-shaped distribution within seven orders of dynamic range of MS signal [24]. The extracted abundance ranges for the 10 most abundant proteins in the cellular fraction include actins, plectin, filamin-A, and histones (Figure 2c, Table S3). In contrast, the 10 most abundant proteins in the conditioned medium include enolases, cystatin-C, complement C3, collagen alpha-1(VI) chain, agrin, and fibronectin (Figure 2d, Table S3).

### 3.1. Proteome Annotation Based on Gene Ontology (GO) Terms

Proteins are synthesized and released by the cells through classical and non-classical secretion pathways. Therefore, it is not surprising to see about 30% of identified proteins are shared between cellular fraction (CF) and conditioned medium (CM) datasets (Figure 1a). It highlights the complexity of protein localization and the dynamic interplay between intracellular and extracellular environments. To gain a more comprehensive view of the proteome differences or similarities between CF and CM, our GO functional classification analyses focused on proteins grouped into three categories: CF, CM, and shared CF/CM, as defined in the Methods section and detailed in Supplementary Material S2 (Table S4). We assessed and compared functional associations based on Gene Ontology (GO) terms that describe the cellular component, molecular function, and biological process for the CF, CM, and shared CF/CM proteome datasets. This analysis identified several significant enrichments of GO terms linked to the MeT-5A proteome (Supplementary Material S2, Tables S5–S7).

#### 3.1.1. GO Cellular Component Enrichment

**Figure 3:**
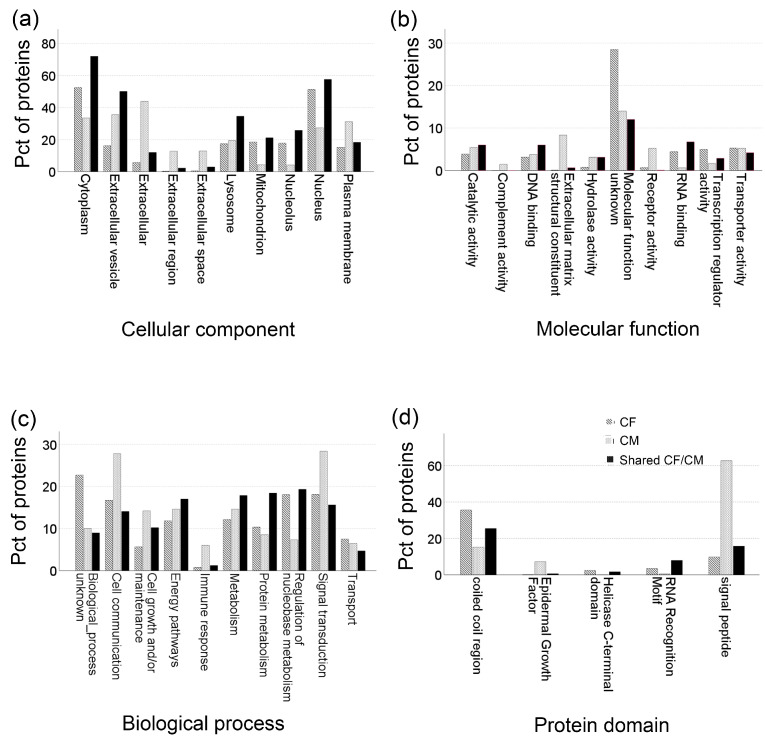
Cellular component, molecular function, and biological process enrichment for distinct protein sets. Proteins uniquely or significantly enriched within the cellular fraction (CF), proteins from the conditioned medium (CM), and proteins shared between both fractions (shared CF/CM). Gene Ontology (GO) enrichment analysis was performed to identify the top 10 most significantly enriched terms within each dataset for three standard GO categories: (**a**) Cellular Component, (**b**) Molecular Function, and (**c**) Biological Process. A fourth analysis, (**d**) Protein domain, identifies top five enriched protein domains. The bar graphs illustrate the percentage of proteins within each dataset that are associated with a specific GO term, and protein domain. Enrichment analysis was conducted using the FunRich tool and FunRich background database, applying a hypergeometric test with a false discovery rate (FDR) correction for multiple testing. The top 10 terms for each category were selected based on an adjusted *p*-value ≤ 0.01. The bar graph’s fill pattern indicates the origin of the protein datasets: cellular fraction (stripes), conditioned medium (dot), and shared proteins (black).

Results of GO cellular component analysis revealed an over-representation of extracellular proteins in CM when compared with the CF and shared CF/CM protein datasets. Moreover, a greater proportion of CM and shared CF/CM proteins showed extracellular vesicles enrichment than CF proteins (Figure 3a, Tables S5–S7). CF and the shared CF/CM proteins are predominantly located within the cytoplasm and nucleus.

Historically, the presence of cytoplasmic and nuclear proteins in conditioned medium was mainly attributed to release from dead cells [25,26]. Under the culture conditions of the MeT-5A cells in this study, the estimated cell death was 5–10% based on the trypan blue staining assay. If cytosolic and nuclear proteins are released primarily through cell death, their relative abundance in the medium would likely be influenced by the estimated percent of cell death. However, out of the 100 most abundant proteins (based on mean normalized PSM) in the cellular fraction (Figure 2c, Table S3) or in the conditioned medium (Figure 2d, Table S3), 53% were found to be unique to either the cellular fraction or to the conditioned medium. These unique proteins are annotated for intracellular or extracellular localization, respectively. The remaining 47% are shared between both and are localized within the extracellular vesicles (EVs). This list includes enolases, and enolases are primarily cytosolic enzymes involved in glycolysis. However, there is a growing body of evidence indicating that alpha-enolase (ENO-1), can be found in other locations, including the cell surface, the nucleus, and EVs, carrying out functions distinct from its glycolytic role [27]. Alpha-enolase’s functions are diverse and heavily dependent on its location inside or outside the cell. For example, on the cell surface, it acts as a plasminogen receptor, which promotes tissue invasion, cancer cell migration, and metastasis [28]. Extracellular ENO-1 is also implicated in various diseases, such as cancer, Alzheimer’s disease, and rheumatoid arthritis [29,30,31]. The abundant presence of enolases in both the cellular and conditioned medium can be attributed to their multiple roles beyond glycolysis and gluconeogenesis. Our data confirms this observation and further demonstrates that the abundant extracellular presence of enolases is not limited to pathogenesis.

**Figure 4:**
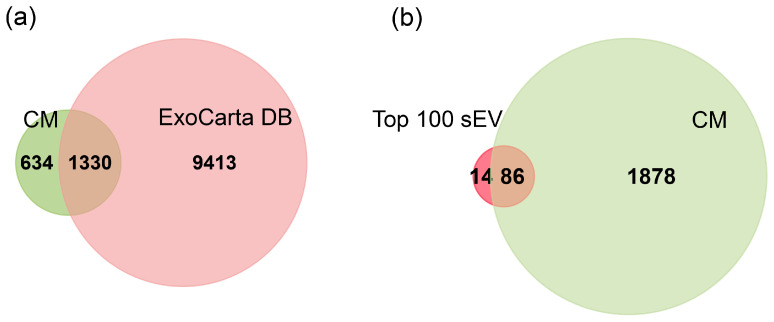
Venn diagrams comparing the conditioned medium (CM) proteome to the ExoCarta database. (**a**) Comparison between the total identified proteins in the CM proteome and all proteins listed in the ExoCarta database (20 March 2025 release). (**b**) A focused comparison between the CM proteome and the top 100 most abundant small extracellular vesicle (sEV) proteins from ExoCarta (20 March 2025 release). Numbers inside the circles indicate the total number of proteins identified in each set (green circle: conditioned medium proteome; other circle: ExoCarta data); the overlapping region represents the number of proteins identified in both datasets.

Enriched localization within extracellular vesicles (EVs) is not limited to highly abundant proteins; approximately 24% of proteins identified in this study are cargo specifically localized to these vesicles (Figure 3a). EVs are specialized vesicles that cells use to release cargo, including specific proteins, into the extracellular space. Of the 10,743 small EV proteins compiled in the ExoCarta database, 1330 were common to both the conditioned medium (CM) proteome and the ExoCarta datasets (Figure 4a). In addition, 86 of the top 100 sEV documented in the ExoCarta database were also identified in the CM (Figure 4b). This substantial overlap, representing approximately 67% of the total proteins identified in our CM proteome, robustly confirms that the identified EV proteins, determined via FunRich Gene Ontology (GO) term enrichment, aligns with established EV origins. The high abundance of these cargo proteins in our study suggests their release into the conditioned medium is primarily driven by EVs and non-classical secretion pathways, rather than cellular leakage.

In addition, protein domain enrichment analysis of proteins in CM and shared CF/CM revealed that approximately 62.7% of CM proteins and 15.75% of shared CF/CM proteins have signaling peptide domains (Figure 3d, Supplementary Material S2, Tables S6 and S7, respectively). This suggests that these proportions of proteins may have been released into the CM through the classical secretion pathway. The remaining proteins, representing the majority of those not released via the classical pathway, are likely shed from the cell surface or released through non-classical secretion pathways, including the exocytosis of secretory lysosomes or EVs themselves [32,33]. EVs and their protein cargo are shown to play a multifaceted role in modulating mesothelial function and transport both within the mesothelium and across it: transport of cargo and signaling molecules, modulation of cellular functions, cross-mesothelial communication, impact on mesothelial to mesenchymal transition, and in promoting disease processes [34,35,36]. Over-enrichment of these cargo proteome clusters in MeT-5A cell culture and conditioned medium perhaps highlights that EVs are integral to mesothelial function and transport, playing a role in both local and long-range intercellular communication. Further research into the precise mechanisms of EVs biogenesis, cargo sorting, and interaction with target cells is critical for a deeper understanding of mesothelial health and disease.

#### 3.1.2. GO Molecular Function Enrichment

The GO molecular function analysis results revealed distinct or shared functional profiles for the CF, CM, and proteins common to both (shared CF/CM) (Figure 3b). Further molecular type annotations mirrored these functional profiles (Supplementary Material S3, Figure S1). Proteins from the CF dataset were linked to diverse intracellular activities, such as RNA binding, translation, and providing structural components for the cytoskeleton and ribosomes. They also displayed various catalytic functions, including ATPase and oxidoreductase activity (Figure 3b, Table S5). Conversely, CM proteins were primarily involved in extracellular functions and cell-to-cell communication. These functions included protease inhibitor activity, receptor binding, growth factor activity, and constituting the extracellular matrix (Figure 3b, Table S6).

The functional roles identified for the CM proteins are highly consistent with the known biology of mesothelial cells. These cells, which line serosal cavities, actively participate in cell communication and interact with the surrounding extracellular matrix. For instance, mesothelial cells regulate fibrin levels by secreting procoagulant and fibrinolytic enzymes, such as tissue factor (TF) and plasminogen activator inhibitors (PAI) [37]. They also produce and respond to growth factors like PDGF, EGF, and TGF-β, which are crucial for regulating their proliferation, differentiation, and tissue repair [37,38]. The presence of proteins involved in extracellular matrix structural constituent within the CM dataset points to the role of mesothelium in producing and maintaining the extracellular matrix (ECM) of the serosal membranes. Additionally, the presence of ECM structural constituents in the CM highlights the mesothelium’s role in synthesizing components like collagen (types I, III, and IV), elastin, fibronectin, and laminin to maintain the ECM of serosal membranes. They also contribute to the immune surveillance function by interacting with various cells, including cancer cells, through cell communication [11,39].

Functional annotation of proteins found in both the cellular and conditioned medium (Figure 3b, Table S7) highlights functions that bridge the intracellular and extracellular environments. These shared activities include enzymatic functions, such as catalytic, oxidoreductase, and hydrolase activities, which point to a dynamic exchange of enzymes across the cellular boundary. Also noted is cytoskeletal modulation, suggesting that secreted proteins influence cellular morphology and motility by interacting with the cell surface.

The functional profile of the mesothelial cell proteome is consistent with the multifaceted roles of these cells within serosal cavities. By illuminating the interplay between intracellular and extracellular protein functions, this analysis offers new insights into mesothelial cell biology, including roles in maintaining tissue integrity, mediating inflammation, orchestrating injury response, and contributing to diseases like fibrosis or cancer.

#### 3.1.3. GO Bioprocess Enrichment

GO bioprocess enrichment analysis of the three proteome datasets (CF, CM, and shared CF/CM) revealed preferential enrichment of specific biological processes (Figure 3c, Supplementary Material S2, Tables S5–S7): The mesothelial medium proteome is more enriched in signal transduction, cell communication, cell growth and maintenance, and immune responses compared to the cellular proteome. This suggests that the proteins involved in these functions are more actively secreted or released into the extracellular environment by mesothelial cells. However, the mesothelial cellular proteome is more enriched in RNA or protein metabolism, regulation of nucleic acid metabolism and DNA replication than the medium proteome. These findings suggest that the mesothelial medium proteome is actively involved in mediating interactions with the extracellular environment and regulating bioprocesses like signal transduction, cell communication, growth, and immune responses. Conversely, the mesothelial cellular proteome primarily focuses on maintaining the cell’s internal metabolic and energy-generating functions.

### 3.2. Protein Domain

Based on functional analysis with respect to protein domains, a key difference was observed between the cellular (CF) and conditioned medium (CM) unique proteomes (Figure 3d, Supplementary Material S2, Tables S5 and S6, respectively). The CF proteome showed a preferential enrichment of coiled-coil domains. These domains are known to be involved in mediating protein–protein interactions and forming stable protein complexes, playing a role in various cellular processes and maintaining the structure and organization of cell machinery [40,41]. In contrast, the CM proteome was significantly enriched for signal peptides. This suggests a higher proportion of CM unique proteins are trafficked through the secretory pathway within the conditioned medium, as signal peptides are typically responsible for targeting proteins to the endoplasmic reticulum for secretion or membrane insertion [42]. Notably, coiled-coil domains were also enriched in shared CF/CM proteome (Figure 3d, Table S7). This indicates the structural importance of coiled-coil domains in both the intracellular and secreted protein populations.

### 3.3. Canonical Pathway Enrichment

We performed canonical pathway enrichment analysis on three datasets (CF, CM, and shared CF/CM) to investigate the distinct and overlapping functions of protein modules. This analysis identified several significant pathways associated with MeT-5A proteome (Supplementary Materials S2, Table S8). By comparing the top ten enriched pathways across these datasets, we uncovered specific functional patterns that reveal the discrete roles of the intracellular and secreted proteomes and how they may be functionally integrated (Figure 4, Supplementary Material S2, Table S8).

**Figure 5:**
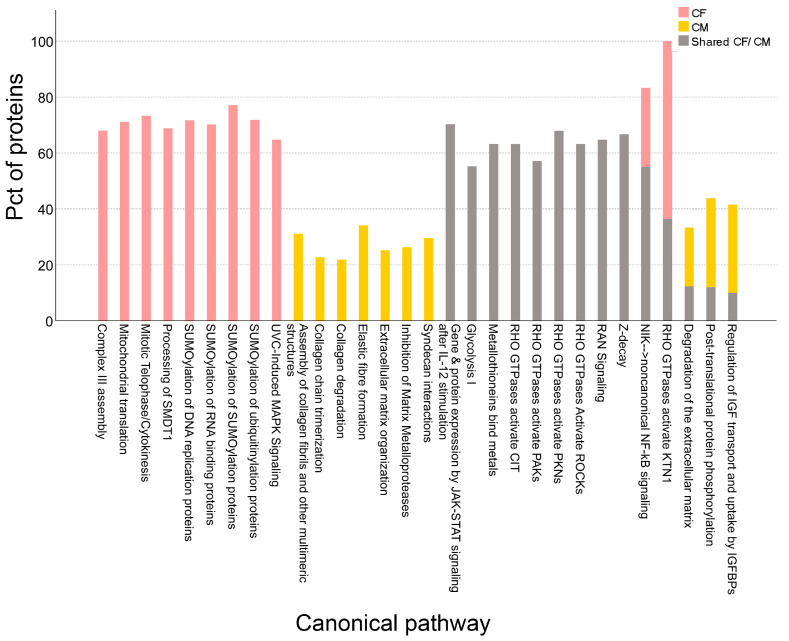
Top 10 enriched canonical pathways identified in three distinct protein datasets. Pathway enrichment analysis was performed using Ingenuity Pathway Analysis Tool with Fisher’s exact test (*p* ≤ 0.01) correction for multiple testing using the false discovery rate (FDR). For each pathway, the bar graph illustrates the percentage of identified proteins within the dataset compared to the total number of proteins associated with that pathway. The bars are colored as follows: uniquely identified cellular fraction (CF, pink), uniquely identified conditioned medium (CM, yellow), and proteins shared between the cellular fraction and conditioned medium (shared CF/CM, gray).

The CF-dataset highlights the core activities within the cell (Figure 5, pink bars). Notably, canonical pathway analysis revealed significant enrichment of processes associated with fundamental internal cellular functions, including post-translational modifications and core metabolic pathways. A prominent finding was the enrichment of pathways for Small Ubiquitin-like Modifier (SUMO) conjugation, reinforcing the intracellular proteome’s critical role in cellular regulation [43].

In contrast, the CM-dataset showed enrichment consistent with its extracellular role (Figure 5, yellow bars). As expected, these pathways were primarily associated with extracellular matrix (ECM) organization and remodeling, a key function of the secreted proteome [43,44,45]. The clear segregation of enriched pathways in the two datasets underscores the distinct biological specializations of the intracellular and extracellular compartments.

The shared CF/CM proteome revealed significant enrichment of several signaling pathways, as shown in Figure 5 (gray bars). A notable finding is that while these pathways operate intracellularly, their activity impacts the protein content of both the cell and the conditioned medium. Two prominent examples are the Rho GTPase and JAK-STAT signaling pathways. Rho GTPase signaling organizes the actin cytoskeleton, which regulates cell shape, migration, and adhesion. This process can lead to the shedding or active secretion of proteins into the extracellular space, thereby altering the conditioned medium’s protein composition. Similarly, JAK-STAT signaling, activated by transmembrane receptors after extracellular stimulation (e.g., IL-12), leads to the nuclear regulation of gene expression. Although the core activities of these pathways are cytoplasmic and nuclear, the resulting changes in protein synthesis can indirectly modify the set of proteins available for secretion [45,46]. The shared proteome also showed enrichment for pathways typically considered restricted to the intracellular space, including Glycolysis, Z-decay, and Ran signaling. This observation is consistent with reports showing that components of intracellular pathways can be released into the extracellular environment [47,48,49]. For instance, glycolytic enzymes are often found in extracellular vesicles (EVs), such as exosomes, where they enable active extracellular metabolic functions, particularly in the tumor microenvironment [47,48]. Similarly, proteins from the Z-decay and Ran signaling pathways are likely packaged and released via these transport vesicles. Further investigation is needed to determine if the extracellular functions of these secreted proteins are the same as or different from their intracellular roles.

Our analysis identified an additional category of pathways with protein components shared between the intracellular and extracellular proteomes (Figure 5, stacked bars). This category includes both proteins found in either the CF or CM alone, and those shared between them. The pathways fall into two distinct types. The first, exemplified by the NIK non-canonical NF-kB cascade and RHO GTPase-activated KTN1 signaling, contains a mix of proteins unique to the CF and those shared across both compartments (Figure 5, gray/pink stacked bars). The second type consists of three pathways predominantly enriched in the CM, which also contain some shared CF/CM protein components (Figure 5, gray/yellow stacked bars). Notably, this second type includes the post-translational protein phosphorylation pathway. While phosphorylation is a classical intracellular signaling mechanism, its role in regulating extracellular matrix interactions is an emerging area of research [50,51,52]. Our data confirms the presence of these phosphorylation pathways in the extracellular fraction, demonstrating that this enrichment is not exclusive to cancer cells, contrary to previous suggestions [53]. This finding advances our understanding beyond specific pathologies, highlighting the existence of complex, bidirectional signaling mediated by proteins that dynamically operate across cellular and extracellular environments.

### 3.4. Shared Proteins Between MeT-5A and Innate Immunity

We compared the mesothelial proteome with the known innate immune-related proteins documented in the InnateDB database (innatedb.ca), accessed and down loaded on 4 September 2024, to identify proteins shared by both datasets. Out of 1045 innate immune-related proteins compiled from InnateDB, 295 were identified as common to both the mesothelial proteome and the innate immune datasets (Supplementary Material S3, Figure S2). The identification of these 295 common proteins suggests a potential link between mesothelial cell function and the innate immune system. InnateDB is a valuable resource for investigating these proteins and their roles in innate immunity. Mesothelial cells, lining various body cavities, play diverse roles beyond simple barrier function, including cytokine production and inflammation mediation [7,11]. The presence of these shared proteins suggests mesothelial cells likely actively contribute to innate immune responses, potentially impacting areas like inflammation, pathogen recognition, and immune cell recruitment.

#### Study Limitations

This study’s comprehensive proteomic analysis, while representing a significant advance for mesothelial cell research, is subject to the inherent limitations of the bottom-up mass spectrometry platform. The digestion-dependent workflow fundamentally precludes a comprehensive analysis of the full range of proteoforms, as the peptides generated lose connectivity to their parent proteins. Consequently, our findings should not be interpreted as a full characterization of PTMs or the precise identification of all protein variants. The use of protein groups to mitigate the peptide ambiguity problem also means that distinctions between related protein species may be masked within our dataset. These limitations do not diminish the value of this foundational dataset but rather define the scope of future, more targeted investigations. Moving forward, the mesothelial cell protein inventory established here can inform the strategic application of advanced proteomics techniques, such as top-down or targeted MS, to resolve protein-level complexity and probe specific biological hypotheses.

It is also important to note that the proteomics profile presented for conditioned medium represents the total protein content of the conditioned medium, encompassing both actively secreted proteins and contaminants released due to an inherent 5–10% level of cell death observed during the culture period. To mitigate the impact of passive release and distinguish the genuine secretome, we implemented a rigorous bioinformatics analysis: comparing the conditioned medium proteins profile and quantitative spectral count against the corresponding cell lysate profile, proteins identified with high enrichment ratio in the conditioned medium compared in the cell lysate were prioritized. Furthermore, we utilized GO cellular localization annotations, cross referenced with the ExoCarta database and analyzed protein domain enrichments to classify proteins as confidently secreted (e.g., signaling peptide bearing proteins) or likely intracellular contaminants. We acknowledge that while these filters help enrich for true secretome components, a minor proportion of contaminants may persist, particularly those with high leakage propensity. Therefore, the data are conservatively reported as the baseline conditioned medium proteome to provide a comprehensive, yet carefully curated, overview for subsequent functional validation analysis studies.

We also acknowledge that merging conditioned medium from different time points provides a comprehensive overview of the secretome but does not capture dynamic, time-resolved changes. A detailed time-course analysis would be an important future direction for this research.

## 4. Conclusions

This study established the initial baseline proteome for the intracellular and extracellular compartments of MeT-5A mesothelial cells, providing a critical reference for in vitro models. The proteomic functional analysis revealed a distinct functional specialization within the mesothelial cell. The extracellular proteome, found in the conditioned medium, is rich with proteins that facilitate cell communication, shape the extracellular matrix, and modulate immune responses, underscoring the mesothelium’s role as a dynamic, interactive barrier. In contrast, the intracellular proteome is primarily dedicated to the core metabolic and energy production pathways essential for cell survival and environmental responsiveness. This division of labor demonstrates the complex balance between internal cellular maintenance and external interactions. A notable finding was the significant number of shared proteins between the cellular and conditioned medium proteins highlighting the need for further investigation into their noncanonical extracellular functions. This foundational data creates a solid basis for future comparative and functional proteomic studies involving this immortalized human mesothelial cell line.

Our identification of a high number of innate immunity-associated proteins further supports the mesothelial tissue’s function as a first-line defense barrier against infection. This information offers significant relevance for researchers using mesothelial models and provides insights that are potentially inferable to in vivo situations. Establishing this baseline proteome paves the way for crucial comparative studies and lays the groundwork for further exploration of these proteins and their immune-related functions.

## Data Availability

The raw mass spectrometry data sets are deposited at URL (ftp://massive-ftp.ucsd.edu/v11/MSV000099866/) (accessed on 10 November 2025) on the proteomeeXchange consortium.

## Supplementary Materials

The following supporting information can be downloaded at: https://www.mdpi.com/article/10.3390/proteomes14010002/s1, Supplementary Material S1: Table S1: Database search results for cellular proteins; Table S2: Database search results for conditioned medium proteins; Table S3: Comparison of top 100 cellular and conditioned medium proteome; Supplementary Material S2: Table S4: List of proteins unique and enriched within the cellular fraction (CF), conditioned medium (CM), and proteins present in both CF and CM (shared CF/CM); Table S5: Gene Ontological annotation results for CF; Table S6: Gene Ontological annotation results for CM; Table S7: Gene Ontological annotation results for shared CF/CM; Table S8: Canonical pathway enrichment results. Supplementary Material S3: Figure S1: Molecular type enrichment for distinct protein sets. Figure S2: Venn diagram comparing the mesothelial cell MeT-5A proteome to the innateDB.
