# Supplementary material for: Proteomics and Bioinformatics Profiles of Human Mesothelial Cell Line MeT-5A
Source: Proteomes. 2026 Jan 4;14(1):2. doi: 10.3390/proteomes14010002 (PMC12821629; doi:10.3390/proteomes14010002)
Supplement: Supplementary file 1 [file proteomes-14-00002-s001.zip › Supplementary Material 3.pdf]

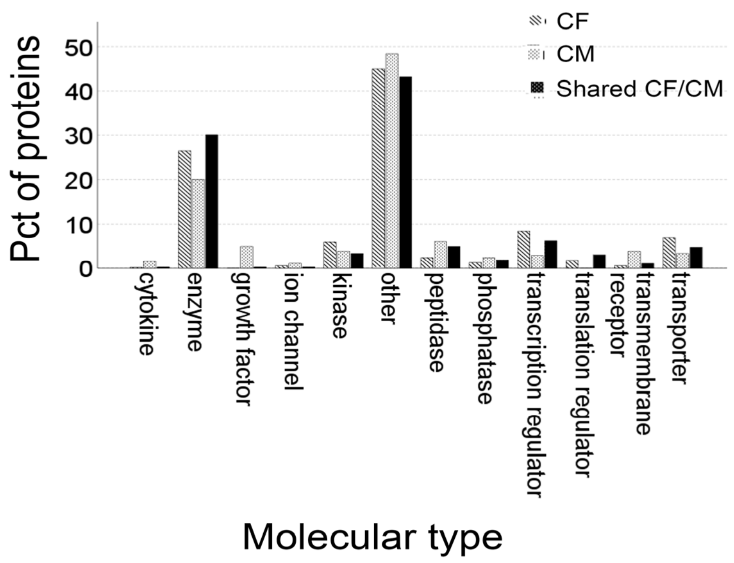


Figure S1: **Molecular type for distinct protein sets.** Proteins uniquely or significantly enriched within the cellular fraction (CF), proteins from the conditioned medium (CM), and proteins shared between both fractions (shared CF/CM).

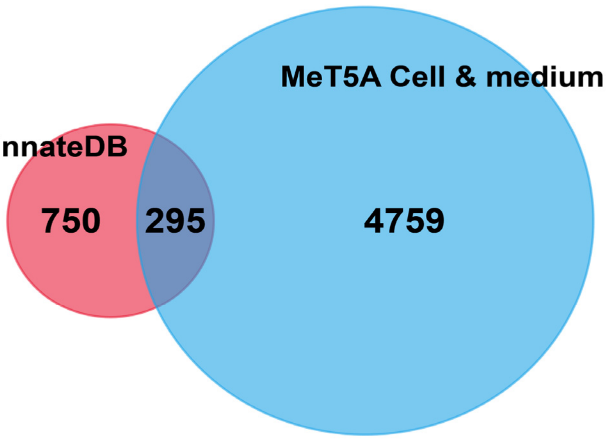


Figure S2: **Venn diagrams comparing the mesothelial cell MeT-5A proteome to the innateDB.** Comparison between the total identified proteins to the known innate immune-

related proteins documented in the InnateDB database (innatedb.ca). Numbers inside the circles indicate the total number of proteins identified in each set (blue circle: total identified proteins in both cellular lysate and conditioned medium; other circle: innateDB); the overlapping region represents the number of proteins identified in both datasets.
